# On the Distribution of Muscle Signals: A Method for Distance-Based Classification of Human Gestures

**DOI:** 10.3390/s23177441

**Published:** 2023-08-26

**Authors:** Jonas Große Sundrup, Katja Mombaur

**Affiliations:** 1 Canada Excellence Research Chair Human-Centred Robotics and Machine Intelligence, Systems Design Engineering & Mechanical and Mechatronics Engineering, University of Waterloo, Waterloo, ON N2L 3G1, Canada; katja.mombaur@uwaterloo.ca; 2 Optimization and Biomechanics for Human-Centred Robotics, Institute of Anthropomatics and Robotics, Karlsruhe Institute of Technology, 76131 Karlsruhe, Germany

**Keywords:** EMG, gesture recognition, dynamic time warping, classification, signal processing

## Abstract

We investigate the distribution of muscle signatures of human hand gestures under Dynamic Time Warping. For this we present a k-Nearest-Neighbors classifier using Dynamic Time Warping for the distance estimate. To understand the resulting classification performance, we investigate the distribution of the recorded samples and derive a method of assessing the separability of a set of gestures. In addition to this, we present and evaluate two approaches with reduced real-time computational cost with regards to their effectiveness and the mechanics behind them. We further investigate the impact of different parameters with regards to practical usability and background rejection, allowing fine-tuning of the induced classification procedure.

## 1. Introduction

Gesture recognition systems are gaining rapid popularity due to a variety of applications, ranging from hands-free computer interfaces in industry applications over human robot interactions up to controlling exoskeletons or smart homes or communicating with humanoid robots. Approaches based on optical sensors [1,2,3,4] have the disadvantage of needing cameras, limiting their use, whereas surface-mounted electromyography-sensors (sEMG-sensors) allow us to measure muscle activity and therefore to build much more portable and embedded sensor systems.

As a significant portion of the muscles required for finger movement is located in the forearm and its muscular signal is indicative for the gesture or finger motion performed by a person. Therefore, an sEMG sensor system on the forearm can be used for gesture classification. In this paper, we will discuss how these muscle signals can be leveraged as a predictor for hand gestures for robust high quality gesture recognition and cluster identification.

Surface-EMG-based methods for gesture recognition have already been proposed by several publications, among them artificial neural networks, to achieve up to 87% accuracy with a single-channel-sensor when attempting to distinguish four different gestures [5]. Generally, black-box neural networks, particularly convolutional neural networks, have emerged in popularity to analyze sEMG gesture data [6,7,8,9,10,11,12,13,14,15,16,17]. A composition consisting of a Bayes classifier and a k-Nearest-Neighbors classifier can achieve up to 93% accuracy for four different gestures with one channel [18]. An increased number of 5-channels for the sEMG sensors combined with acceleration data can achieve accuracies around 98% using a decision tree constructed of several types of classifiers, but with sEMG-measurements only this yields just 79.7% (average) or 85.8% (median) [19]. This has been surpassed using an 8-channel sEMG sensor by [20], yielding accuracies above 90%, depending on the number of different gestures to distinguish, and 97% can be achieved with gel electrodes with four sensors [21], but their practical use is limited due to the requirement of wetness on the electrodes. An approach similar to ours, with the same hardware, but without consideraticon of normalization and condensation and with only five instead of seven gestures, has been proposed by [22], resulting in an average of 89.5% accuracy, surpassing the Myo’s built-in system which they quantify as 83.1% accurate. Further recent experiments by [23] with the Myo Armband and deep and double-deep Q-networks could achieve up to 90.37%±10.7% accuracy, and 97% on six gestures with the same hardware as we use could be achieved using spectral collaborative representation-based classification [24], which is a component-based identification approach. A combination of Dynamic Time Warping and Bayes classification resulting in a score-based system has been shown to yield an average accuracy of 95% with a comparatively large gesture vocabulary of 19 gestures [25].

Furthermore, the classification procedures in these papers do not provide an inherent property to distinguish gestures-to-be-classified from background noise and/or gestures not in the classification pool, with the exception of [22], who, however, did not address this question either. Most publications [5,18,21] only distinguish between predefined gesture recordings and do not address the issue of distinction of notable gestures from background and arbitraty motion at all. In [19], the distinction of active and non-active periods in the EMG signal was proposed, which is capable of distinguishing between gesture and resting position, but does not give a clear distinction between desired gestures and arbitrary motion or undesired gestures. Also, they do not provide a clear understanding how the differentiation is achieved, limiting the possibility to intelligently decide on a robust set of features that is clearly distinguishable and easy to both differentiate and detect.

We present our approach using 8 sEMG sensors surrounding the forearm. This makes the approach more resilient to misplacement of the electrodes as we acquire a more complete measurement of the muscle activity around the entire forearm. In addition to that, different muscle areas playing together for a gesture can be easily correlated to a characteristic gesture signature. Furthermore, we do not abstract the signal into characteristics but instead perform the classification directly on the EMG signals, yielding an intuitive way of measuring similarity as well as defining criteria for it to improve accuracy. Fundamentally, in this paper we will address *three key questions*:

- Is the classification accuracy sufficient for use of such a system in practice?
- What is the underlying reason this classification exhibits this classification performance?
- How can this classification performance be retained reducing both computational and user effort?

**Figure 1:**
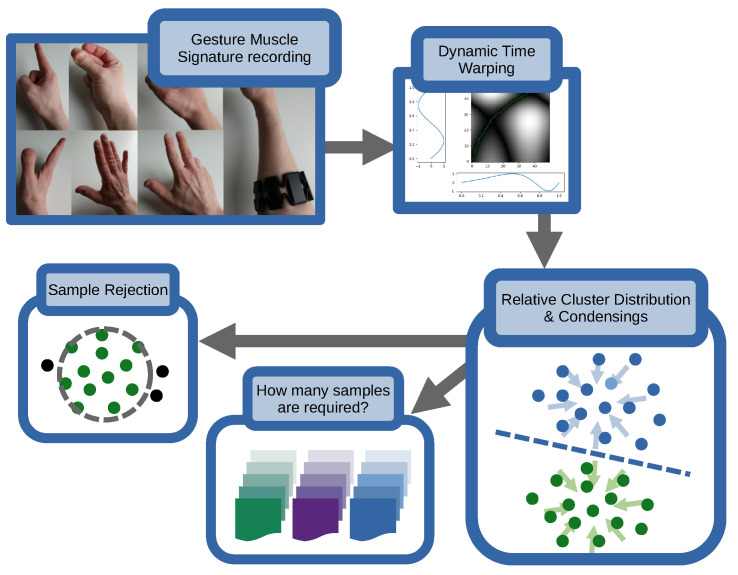
Visualization of the work presented. The (**top left**) indicates gestures recorded, which are compared via Dynamic Time Warping in the (**top right**). This then allows the assessment of cluster distributions and cluster condensing (**bottom right**), which lays the foundation for investigations such as the number of samples required and sample rejection (**bottom left**).

In Section 2, we first lay out the theoretical foundation this work is based on, followed by the experimental setup in Section 2.1. We will review the fundamental underlying methods in Section 2.2 and discuss methods of dataset condensing in Section 2.2.3, the results of which we will see initially in our initial feasability assessment in Section 3.1. We will then investigate the question of how to quantify sample distributions in non-metric space in Section 2.2.4, with applications of this in Section 3.2, and will revisit this throughout the rest of the paper before proceeding to lay out an approach for acceptance limitations for real-world datastream classification in Section 2.2.5 with corresponding results in Section 3.4. Finally, we will investigate the question of transfer of data in this framework between participants, method-wise in Section 2.2.6, with Section 3.5 listing the corresponding results. Afterwards we will discuss the results presented and conclude this investigation, including an outlook. The general flow of the paper is also visualized in Figure 1.

## 2. Materials and Methods

We will review both the form and structure of the acquired data that was used for this research as well as the methodology. Starting with the data, we will review the base gesture set as well as the hardware used to generate the underlying data, as well as the acquisition procedure.

Afterwards, we will investigate the underlying methodology to compare gesture recordings under consideration of time-shifts in the underlying motion. In doing so, we will briefly review Dynamic Time Warping and k-Nearest-Neighbors as the foundational methods of this investigation and methods to investigate structural distributions within non-linear, non-metric time-adjusted space. We then proceed in proposing condensing methods to reduce the dataset size without compromizing the dataset structure as well as investigating methods to be able to reject data that does not fit any of the prerecorded gestures in the set. Finally, we will introduce approaches that might allow transferring highly indiviualistic data such as EMG signatures from one participant to another.

### 2.1. Base Gestures and Datasets

**Figure 2:**
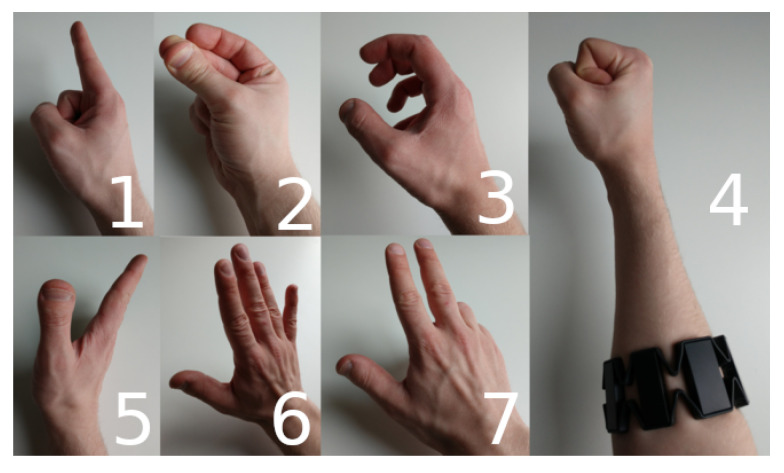
Gestures used to test our classification procedure. Top row, left to right: point, snap, open. Bottom row, left to right: wave out, wave, count. Right column: fist with Myo Armband attached to the forearm.

For our analysis we selected seven gestures, also depicted in Figure 2, using the Myo Armband [26]:

1. **point:** Pointing the index finger outwards.
2. **snap:** Finger snapping with thumb and middle finger.
3. **open:** Opening the hand from a fist position, reverse of *fist*.
4. **fist:** Making a fist from an open hand position.
5. **wave out:** Waving perpendicular to the hand surface.
6. **wave:** Waving in the plane of the hand surface.
7. **count:** Opening the hand from a fist position, one finger at a time.

When selecting these gestures, we took rough inspiration from the gestures the Myo Armband’s own proprietary classification system promises to be able to classify, but both slightly increased the number for good measure and also some gestures that seem fairly similar, such as point and wave out, were be included, which will be of particular interest later on.

The Myo itself consists of eight surface-mounted electromyography-sensors in a circular arrangement around the forearm and is worn where muscles are thickest, gathering charges emitted by the underlying muscles upon activity. The data acquisition frequency of those electrodes was 200 Hz. This yields eight synchronous activity measurements over time, one for each sensor, for the covered muscle parties, resulting in a timeseries per gesture of dimension 8×L where *L* is the length of the recorded gesture. As the movements of the fingers are strapped to this muscle region via tendons, the lower arm muscle activity can be leveraged as a proxy for finger movement. It is also equipped with a multi-axis IMU, which will not be used but has been used in various circumstances, for example, for sensor fusion [27].

Although the Myo armband was discontinued by the manufacturer [28], our approach is independent of this specific hardware, and it just happens to provide an easy-to-use sEMG sensor setup for our measurements, which made it popular for a variety of applications, such as post-stroke muscle strength assessment [29] and pose [30] and pointing [31] detection, being used to control external systems [32] and used as a datasource for method research, such as working on feature extraction [33]. It might be transferrable to other devices of this kind, for example those listed in [34].

#### 2.1.1. Data Acquisition Procedure

We used the Myo armband to gather electromyography data of the aforementioned gestures with 100 measurements each, summing up to 700 samples alltogether, on two different participants without repositioning the Myo. The participants were instructed to perform the previously mentioned gestures on their own and were provided with software to autonomously record the described number of samples without supervision, representing a situation as it would occur assuming the participant would autonomously operate the device for their own private or professional use. This implies that the data was recorded within the frame of a subject’s natural preference, increasing the chances of consistency, but also implies that the samples are not all of equal length. Different gestures do have a different natural pacing, and also within the same gesture the start and end timing can be slightly off, requiring the underlying method to address this in one way or another.

The participants were allowed to make the recordings unsupervised at their own pace after being preinstructed.

#### 2.1.2. Resulting Datasets

Two datasets were acquired per participant: The “singleday” dataset is comprised of 100 samples per gesture without removing or changing the seating of the Myo Armband, resulting in 700 gesture samples at the same location. The “multiday” dataset, in contrast to that, recorded 20 samples per gesture per seating, and the Myo was taken off after each recording. As participants were instructed to seat the Myo Armband identically for each recording session, this leads to variations that resemble those naturally occurring when a participant operates such a device autonomously during day-to-day operations or life. Participants were instructed to take and took several days to record this dataset. A total of 5 seatings was recorded for the “multiday” dataset.

All recording sessions are identical except for sample numbers. However, to preserve sample number balance (and consequentially an equal weighting of different random exact positionings of the different sensors), the singleday dataset is not merged into the data of the multiday dataset when performing analyses, as it would correspond to a day of disproportionally increased sample count, posing a risk of skewing the results. Instead, when compared to the multiday dataset serving as a high-sample-count location-variation-free reference dataset for some parts of this investigation.

In addition to this, participants were requested to record a reduced number of samples with maximum voluntary contraction, which we refer to as “powergrip” in this investigation, that will be used when investigating transferability of recordings between subjects.

**Figure 3:**
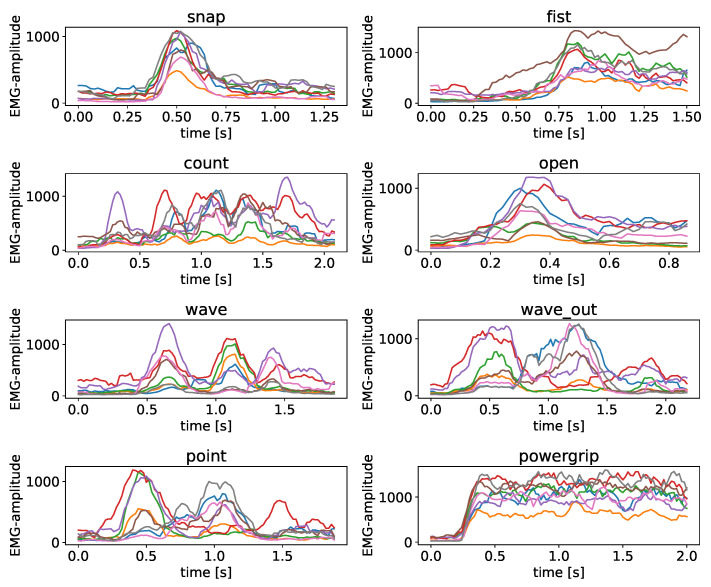
Sample recordings of the seven gestures as well as the powergrip as an 8-dimensional EMG recording. As we are only performing a comparison analysis here, the *y*-axis depicts the intensity units returned directly by the Myo hardware, and each line corresponds to the output of one of the 8 sEMG sensors that provide simultaneous measurements, color consistent over different plots.

Each dataset consists of an 8-dimensional time series per recorded sample. An exemplary excerpt of the acquired data can be seen in Figure 3. In total, two subjects were included in the analysis, one with a more well trained muscle structure around the forearm, and one with a less trained muscle structure, to investigate the impact of muscle size with respect to the findings presented here with a high sample count. The exact numbers of seatings and samples acquired can be found in Table 1.

A similar dataset has been published by [35], with both more participants as well as more gestures but only three days and no equivalent to the statistical size of the singleday dataset. Furthermore, this dataset was recorded with lab-equipment and under lab conditions, making it extremely interesting for investigations of this kind as well, but missing the effect of unsupervised, purely user-operated recordings that characterize our recordings specifically, with the according consequences in practical application.

### 2.2. Time-Adjusted Comparison of EMG Signatures

To perform high quality gesture recognition, two techniques will be combined: the Dynamic Time Warping (DTW) technique to establish a distance measure for recorded muscle signals and, subsequently, the k-Nearest-Neighbors (kNN) algorithm to determine similarity of recorded data to prerecorded reference gestures using this distance measure.

#### 2.2.1. k-Nearest-Neighbors Algorithm

The kNN algorithm [36] performs a classification of a given datum by estimating its distance to the reference population of data points. Its class is then determined by the class that represents the majority of its *k* nearest neighbors for a given distance function. A classical choice for the distance function is the euclidian distance; however, we use Dynamic Time Warping, reviewed hereafter, as a much more promising alternative for our use case.

#### 2.2.2. Dynamic Time Warping (DTW)

Dynamic Time Warping (DTW), originally taken from the field of speech recognition [37], is a technique to align two time series that are similar in shape by nonlinearly deforming the time series in time. Comparing these now aligned time series results for our type of data in a much more reasonable distance measure we call the DTW distance. For this reason, it is a powerful and consequentially popular technique in the field of signal processing in a variety of applications, such as manufacturing [38], recurrent pattern tracking [39], oilfield development [40], and the detection of heart rythm disturbances [41].

To obtain a Dynamic Time Warping distance estimate, two sequences $X=(x_{1},\cdots ,x_{N}),$ $Y=(y_{1},\cdots ,y_{M})$ from a feature space *F*, s.t., $(x_{n},y_{m})\in F\times F$ with n∈[1,N]⊂N, m∈[1,M]⊂N, are compared. The warping path is then a sequence $p=(n_{l},m_{l})$ of length *L*, mapping elements from *X* and *Y* onto each other, with $p_{1}=\left(1,1\right)$ and $p_{L}=\left(N,M\right)$ to ensure the endpoints of *X* and *Y* match and respecting monotonicity ($n_{1}\leq \cdots \leq n_{l}\leq \cdots \leq n_{L}$ and $m_{1}\leq \cdots \leq m_{l}\leq \cdots \leq m_{L}$ for 0≤l≤L for each sequence of tuples (n,m)) which is required to ensure that sequences will not be warped backwards. We further define a cost function $c:F\times F\to R_{\leq 0}$ that determines a pairwise cost between points of two sequences. This then allows us to associate a cost with each warping path as $c_{p}\left(X,Y\right)=\sum_{l=0}^{L}c\left(x_{n_{l}},y_{m_{l}}\right)$. An *optimal warping path* $p^{*}$ is then the warping path with the mininimal associated cost, i.e., $p^{*}:=\min_{\left(p\right)}\left(c_{p}\left(X,Y\right)\right)$ and the DTW distance of those two series is then $c_{p^{*}}$. DTW can be used to implement a kNN-classifier for time series data that can yield state-of-the-art classification performance and has been used this way in the past in a variety of applications [42,43].

DTW is a reasonable choice for signals like the one we are using because we want small distances between recordings of gestures if it is the same gesture, even if the time taken for or the relative timings within the gesture were different. Classical comparison of the signal could yield high differeces as corresponding key characteristics if the signals are offset to one another in time or the signals are offset from the start of the recording, especially when classifying on a live datastream without clear distinction of beginning and end of a gesture. DTW is specifically designed to realign these kinds of signals with one another.

For our analysis, we limit the step size the DTW procedure can take as

(1) $$p_{l+1}-p_{l}\in \left(1,0),(0,1),(1,1\right)\;\mathrm{for}\;l\in \left[1,\cdots ,L-1\right]$$

to limit deformation of time series to a physically reasonable amount as well as to ensure monotonicity of the resulting warping path.

This approach, however, still exhibits a bias towards longer sequences: When we compare two time series *X* and *Y* of length *N* and *M*, their cost matrix will be very slim on one side if $N_{1}>>M$, whereas the cost matrix will be approximately square for $N_{2}\approx M$. This results in the cost matrix of $X_{2}$ and *Y* containing a lot more fields the warping path could run through than the cost matrix of $X_{1}$ and *Y*, even though $X_{2}$ and *Y* might intuitively be substantially closer to each other, which in turn biases the DTW distance to prefer shorter sequences. To correct for this, we will normalize each DTW distance by

(2) $$\frac{1}{\sqrt{N^{2}+M^{2}}}\;,$$

which corresponds to a measure of the diagonal of the cost matrix, rescaling the computed DTW distance to one computed on an approximately square, unit-length matrix.

#### 2.2.3. Condensing

The methods presented so far operate on the full dataset. While this is effective, as we will see when discussing the results of this, it comes with a noticable computational cost. These costs can be alleviated by accelerating the DTW itself, either algorithmically [44,45] or by employing specialized hardware [46], or by reducing the necessary number of computations. We propose two condensing methods that will accomplish the latter by reducing the size of the dataset, consequentially reducing the computational cost incurred.

We propose two condensing methods, a one-step as well as a two-step procedure: Firstly, the one-step procedure will select a *representative sample* from the cluster. We define the representative sample to be the sample whose median distance to all other samples in the cluster is smallest, i.e., for a cluster of gestures of the same kind *G*, we select the gesture $g_{i}$ such that

(3) $$\min_{g_{i}}med\left({\left\{DTW\left(g_{i},g_{j}\right)\right\}}_{g_{i},g_{j}\in G,i\neq j}\right)\;.$$

For the two-step procedure, the first step is the identification of a representative as described above. This representative then functions as the initial guess for a DTW barycenter averaging procedure as proposed by [47], which attempts to find an artificial sequence (not just a chosen representative) that is closest to all elements in a sequence cluster but not necessarily identical to any of them. By averaging an entire cluster with this initial guess, we can find a representation that is, as we will later see, better suited to represent said cluster.

We will, particularly in figure legends, refer to the one-step condensing procedure also with the abbreviation “rep”, while the two-step-procedure will also be referred to under the abbreviation “dba”. Should operations on the uncondensed dataset need to be differentiated from the two condensing methods, they will also simply be referred to with “dtw”.

#### 2.2.4. Cluster Distance Distributions

To assess the distributions of clusters relative to each other, we will define two kinds of distributions on a set of gesture clusters $G_{k}$: The set of *intracluster distances* $\delta_{k}$, which denotes the distribution of DTW distances between all samples within the same cluster, i.e.,

(4) $$\delta_{k}=\left\{DTW\left(g_{i},g_{j}\right)\forall g_{i},g_{j}\in G_{k},i<j\right\}\;,$$

as well as the sets of *intracluster distances* $\Delta_{k,l}$, which denote the set of distances of every sample of cluster $G_{k}$ to every sample of cluster $G_{l}$, i.e.,

(5) $$\Delta_{k,l}=\left\{DTW\left(g_{i},g_{j}\right)\forall g_{i}\in G_{k},g_{j}\in G_{l}\right\}\;,$$

This will yield an approximation of the width of a cluster $G_{k}$ via $\delta_{k}$ as well as the distance and overlap between two clusters $G_{k}$ and $G_{l}$, both of which represent all samples of a specific gesture each via $\Delta_{k,l}$.

#### 2.2.5. Acceptance Limitations

To extend this approach to a real-time gesture detection system, one component is missing: differentiating between an actual gesture we want to detect and background noise in the live signal feed. Achieving this folds natively into our proposed method: We introduce a proximity constraint, i.e., a sample we want to classify as a gesture *g* must be closer than a certain distance *d* to the cluster it becomes associated with to be accepted and classified. If this is not the case, we reject the sample for classification. Let

- $\hat{g}$ be the number of samples correctly classified;
- $\tilde{g}$ be the number of gestures classified and not rejected;
- $\#G_{k}$ be the total number of samples of kind *k*.

We define three properties for any given distance cutoff *d*: The **total accuracy**, which we define as the percentage of samples correctly identified as $g_{i}$ against the number of gestures that should be classified as gesture of kind $g_{i}$, i.e.,

(6) $$\alpha =\frac{\sum_{\hat{g}}1}{\sum_{k}\#G_{k}}\;,$$

the **classification accuracy**, which we define as the percentage of samples correctly classified among all samples accepted for classification, i.e.,

(7) $$\beta =\frac{\sum_{\hat{g}}1}{\sum_{\tilde{g}}1}$$

and the **rejection rate**, i.e.,

(8) $$\gamma =\frac{\sum_{k}\#G_{k}-\sum_{\tilde{g}}1}{\sum_{k}\#G_{k}}$$

These properties allow an assessment of the suitability of a given value for the acceptance distance *d* for the stabilization of a specific classification task at hand. The choice of *d* also allows the tuning of the system for different purposes, such as a stricter acceptance distance at the cost of a higher rejection rate, when accuracy is crucial, for example for operational purposes, or a more lenient approach in personal applications, where misclassifications can be more acceptable.

#### 2.2.6. Inter-Participant Assessment

To be able to transfer samples from one participant to another despite physiological differences, we propose a normalization approach: By leveraging the data of maximum voluntary contraction, we can estimate the maximum signal strength $s_{max,c}$ a person can exert on the position of sensor *i*. Consequentially, we define two normalization procedures, the first one being the normalization of all sensors by the average maximum signal strength exerted:

(9) $$g_{i,c,norm}:=\frac{g_{i,c}}{{\bar{s}}_{max}}$$

This way, the effect of specific pronounced muscle groups is damped. Alternatively, we propose a sensor-specific normalization by normalizing every of the eight sensor component *c* by its individual maximum signal strength:

(10) $$g_{i,c,norm}:=\frac{g_{i,c}}{s_{max,c}}$$

This will better reflect the individual physique of the participants and might allow to better address inhomogeneities in the muscular structure of individuals.

### 2.3. Analyses Conducted and Dataset Splits

We will conduct a total of five analyses, most of which will contain a split of the data into reference data and test data.

**Figure 4:**
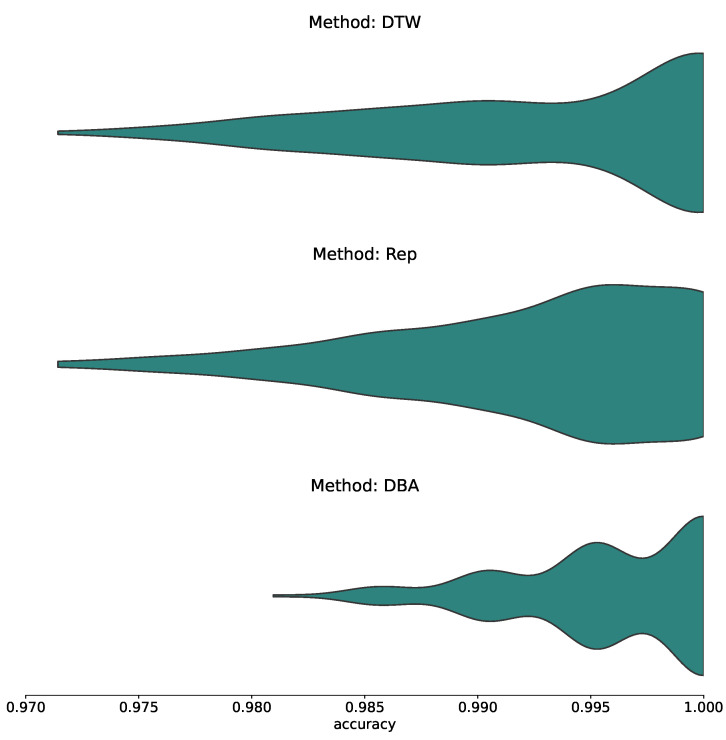
Distribution of accuracies for uncondensed (DTW) and condensed with a selected representative (Rep) as well as barycenter condensing (DBA). The accuracy results of both participants are merged for easier display.

Firstly, to assess the capability of the different methods, we randomly split the singleday dataset of both participants 400 times each into 70% reference data and 30% test data. On each of these 400 resulting reference datasets per participant, we peform both condensation methods per gesture, yielding two condensed datasets and the uncondensed one. We then proceed to run a k-Nearest-Neighbors classification with k=1 to classify the samples in the test data. For the condensed methods, k=1 is required as we condense every gesture cluster down to an indvidual representative; for the uncondensed dataset, it is a choice we made for consistency over the three methods. The distribution of resulting accuracy estimates for each of the different approaches is depicted in Figure 4 for the three methods alongside each other.

Secondly, we will investigate the question of cluster distributions relative to each other. For this analysis, we do not need to split the data as we want to investigate fundamental properties and hence use the full multiday dataset. The multiday dataset will move the relative distribution of clusters closer to a real-life application, hence, we use this for this analysis.

Thirdly, after establishing techniques to improve both classification performance in the form of condensation as well as classification accuracy in the process, there are two more aspects to address: Obtaining a dataset of 700 gestures, while desirable for a proper statistical analysis, is a rather tedious process that will not carry over to user adoption in practice. Hence, we need to investigate how many samples we actually need to provide this level of classification accuracy.

To do so, we will primarily employ the multiday dataset, which exhibits, by virtue of variability in sensor location, a higher spread between samples, consequentially yielding the more conservative constraint onto the data and also likely better describing the actual usage pattern in practice. We will split the dataset into 1 day of test data and 4 days of reference data, each day with 20 samples each. This way, we retain one sensor localization separate from the other, which will directly represent practical applicabiltiy, as in practice a freshly put-on sensor armband will have a close but new set of specific positions. To assess the necessary number of gestures we would need, we will take an increasing number (but the same per day) of samples from the different reference set days and perform a classification experiment. We will discuss the results of this analysis in Section 3.3.

Fourthly, we will investigate the effect of distance limitations for classification acceptance. Here, we will merge the multiday dataset into one dataset and will divide the samples into 10 different splits, 70% reference data and 30% test data each, for each tested cutoff. We will differentiate between the three properties introduced in Section 2.2.5, total accuracy on the whole dataset, classification accuracy and rejection rate. We will then test different cutoff distances *d*. Specifically, we choose the cutoff distances in steps of percentiles of the intracluster distance distribution data δ specific for each gestures, starting at the 5th percentile, and then continuing with the 10th, 15th and so on until the full 100th percentile, including the furthest outlier of the specfiic reference data for the specific gesture in question as well. We compute these percentiles on a per-cluster basis and then aggregate of the resulting total and classification accuracies as well as the rejection rates. We will repeat the analysis with splitting the data by day, with three days constituting the reference set and the remaining two constituting the test set.

Fifthly and finally, we will investigate the transferability of data between participants. This again, similar to the second investigation, will be conducted on the full, non-split dataset, as we are again interested in fundamental properties of the data instead of classification performance under specific circumstances. To focus on the fundamental question of transferability, we use the singleday dataset for this application to remove the localization noise the multiday dataset contains. The type of dataset used for each individual analysis as well as the split between reference and test data (where applicable) is summarized in Table 2.

## 3. Results

In this section, we will first investigate the fundamental question of accuracy of the uncondensed dataset compared to applying condensation. We will further investigate the distribution of gesture samples in non-metric, non-linear time-ajdusted space for both participants. Afterwards, we will investigate the impact of reducing the size of the underlying dataset in consideration of practical applicability as well as acceptance limitations for rejecting sensor readings not included in the gesture set used.

### 3.1. General Accuracy Estimates and the Accuracy Impact of Condensing

The first thing we find is an extremely strong accuracy reading, implying that the method is extremely capable of yielding accurate classifications of the underlying dataset, even when, as we will see in a moment, some parts of the dataset exhibit significant similarities. This is also mostly independent of the condensation applied; particularly, the two-step barycenter condensing exhibits even stronger accuracies than the uncondensed set, whereas the one-step condensation procedure yields an ever so slightly reduced accuracy distribution. Consequentially, this shows that both the approach yields the desired result not only on the raw data but also on the computationally optimized variant in the form of condensing, having direct positive implications for computational cost and hence practical use.

### 3.2. Cluster Distance Distributions

**Figure 5:**
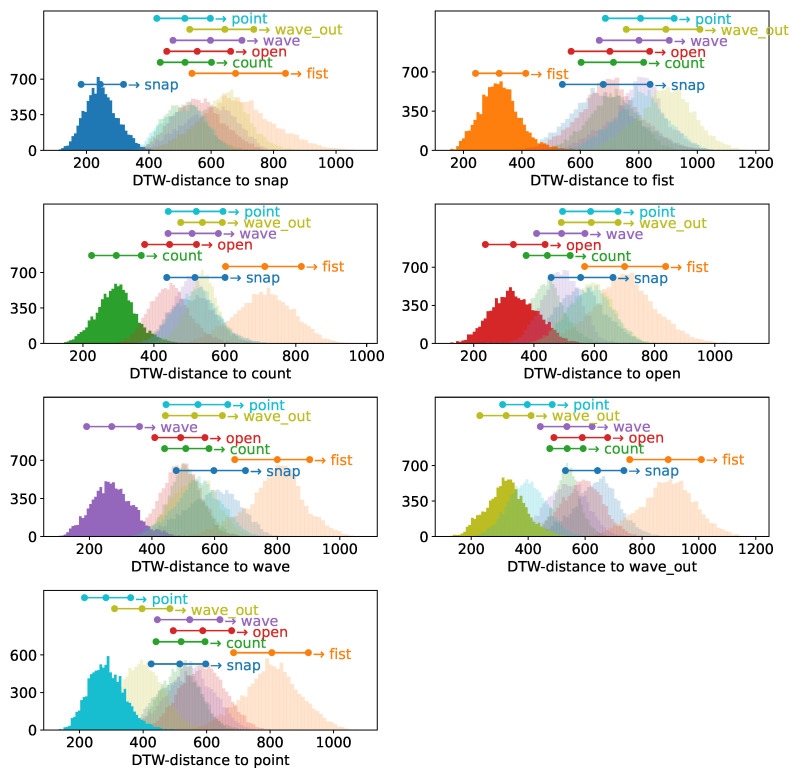
Sample-wise DTW distances of all samples of a given base gesture to all other samples of this gesture within the *multiday* dataset of subject A, depicted in strong colors, as well as to all samples of all other gestures present in the dataset, depicted in different shades. Above the displayed distance distribution histograms, each gesture is annotated with a three-point horizontal bar, indicating the location of the 10th, 50th and 90th percentiles of the corresponding distribution. The individual days of the *multiday* dataset in question were merged into one dataset before analysis.

**Figure 6:**
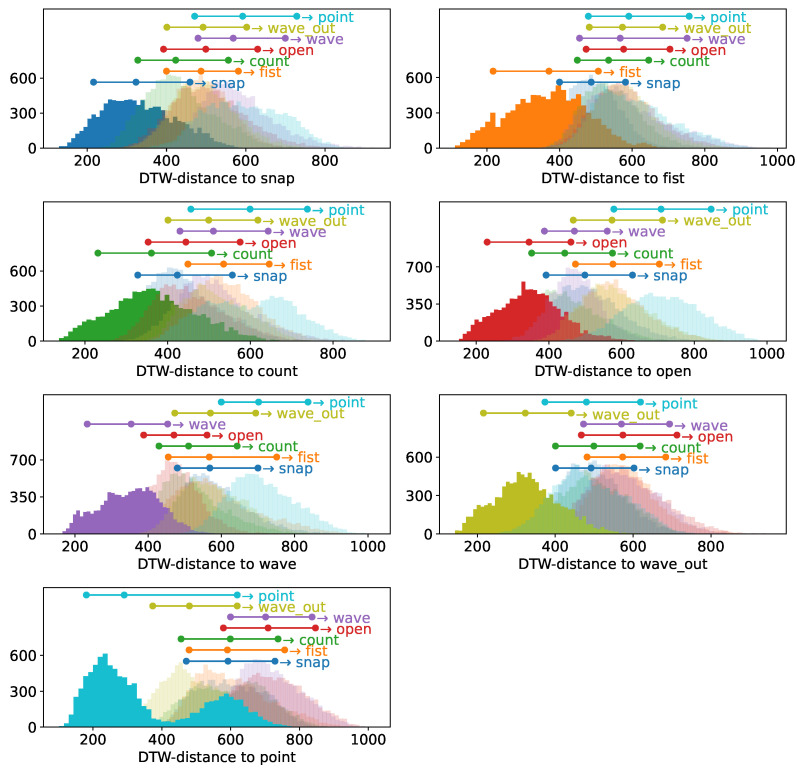
Sample-wise DTW distances of all samples of a given base gesture to all other samples of this gesture within the *multiday* dataset of subject B, depicted in strong colors, as well as to all samples of all other gestures present in the dataset, depicted in different shades. Above the displayed distance distribution histograms, each gesture is annotated with a three-point horizontal bar, indicating the location of the 10th, 50th, and 75th percentile of the corresponding distribution. The individual days of the *multiday* dataset in question were merged into one dataset before analysis.

While the absolute accuracy distribution implies a fundamentally working method, it only allows for a rather superficial explanation of why. Why, however, as the best of all questions, is relevant to both understanding the principles underlying these strong results as well as for making informed decisions of how to further develop such a system. To hence understand this, we will compute the inter- and intracluster distributions as described in Section 2.2.4 for each gesture involved for both participants. We depict the intracluster distribution for each gesture in strong color and each intracluster distance corresponding to this gesture in shades in Figure 5 as well as Figure 6, including markers for each distribution indicationg the 10th, 50th, and 90th percentiles as three dots of corresponding color connected with a bar. Allowing us to still asses the relative distribution of clusters in a space that does not abide by metricity, we clearly see how for participant A the intracluster distance distribution δ is strongly separated from the other clusters for almost all gestures. With one exception, the 90th percentile of all distributions δ, which can be interpreted as a measure of the diameter of a cluster even though metricity is not provided in this space, is smaller than (depending on the case noticably smaller than) the 10th percentile of the corresponding intercluster distances Δ, which can be interpreted as the distance between the outer layers of the two clusters. This means that we indeed find a strong spatial separation between the clusters, explaining why we find such strong accuracy distributions in the previous section. The one exception to this are the gestures “point” and “wave out”, which highly resemble each other, just the number of fingers used in the motion differs. Here, we find a smaller separation between those two clusters, but we still find the distance of the outer layers being larger than the 50th percentile of each δ.

Given the symmetricity of Dynamic Time Warping, this is a phenomenon unsurprisingly observed in both cases, albeit δ for the two gestures slightly differs.

Transitioning to the participant with less strong muscular physique, we do not find such a clear picture, but the fundamental phenomenon that δ for each gesture is smaller than their Δ distributions still stands. In most cases, the 90th percentile of the different Δ distributions is still above the 50th percentile, indicating that the cluster overlap is still slim, particularly considering that the 50th percentile, which describes cluster diameter, would be applied from the center outwards for a distance estimate. This is another strong indicator explaining the excellent performance we have found in the previous section. In addition to that, we find that this participant does not exhibit a similarly pronounced behavior between “point” and “wave out”; instead, it is more similar to the rest of the set, opening the possibility that this effect might not only be explained by the similarity of the two gestures but also by specific patterns of muscle activation that might be less pronounced even on more muscular physiques.

**Figure 7:**
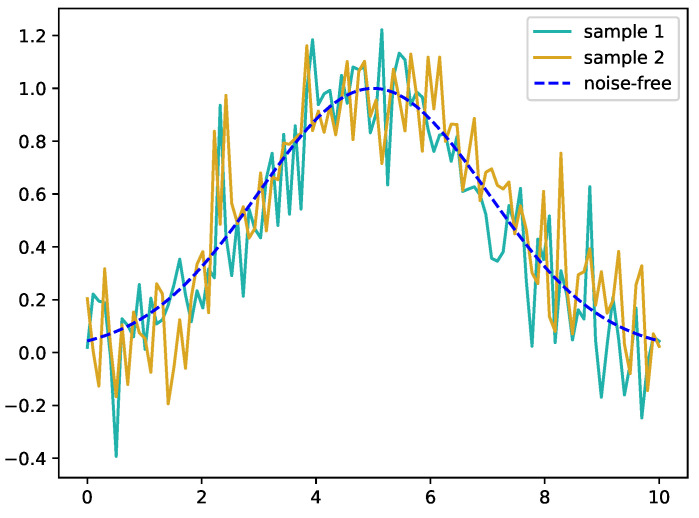
Two samples with different noise patterns applied to the same noise-free base curve, indicated in dashed blue, with μ=5 and κ=2. The applied noise pattern was generated by drawing samples from a zero-centered normal distribution with a standard deviation of σ=0.15.

**Figure 8:**
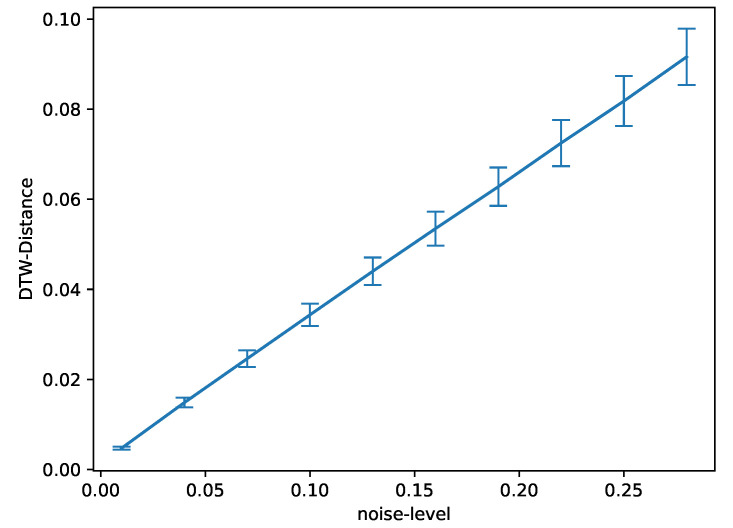
Statistics on DTW distances between two samples based on the base-curve that was depicted in Figure 7 for different amplitudes σ of the noise. The procedure was repeated 1000 times; the error bars indicate the standard deviation of the resulting set of distances per noise level.

The reason why this effect is less pronounced on the subject with less pronounced muscular physique can be easily explained by properties of our distance assessment, namely the dependence of the distance measurement on the signal-to-noise ratio. To show this, assume we have the same curve with two different noise patterns applied to it, as depicted in Figure 7. While both are the exact same signal (hence Dynamic Time Warping should not produce any warping and the DTW distance should be 0), computing the actual DTW distance will be larger than zero due to the noise affecting the procedure. To quantify this effect, we generate two noise patterns to compute two identical but differently noisy sequences 1000 times for different levels of the generated noise and compute the corresponding DTW distance between the two. We find a clear linear dependence of the DTW distance on the noise-level, as depicted in Figure 8, which leads to the conclusion that the higher the level of noise compared to the signal, the more the distributions δ and Δ are washed out by this noise. As the level of noise introduced by the acquisition hardware is similar, given it is the same acquisition hardware, the signal-to-noise ratio is dominated by the signal amplitude, which for less pronounced muscular physiques is lower, and hence the signal-to-noise ratio is equally lower, increasing the effect of noise on the signal on the resulting distributions.

### 3.3. Constraining Reference Data Requirements

**Figure 9:**
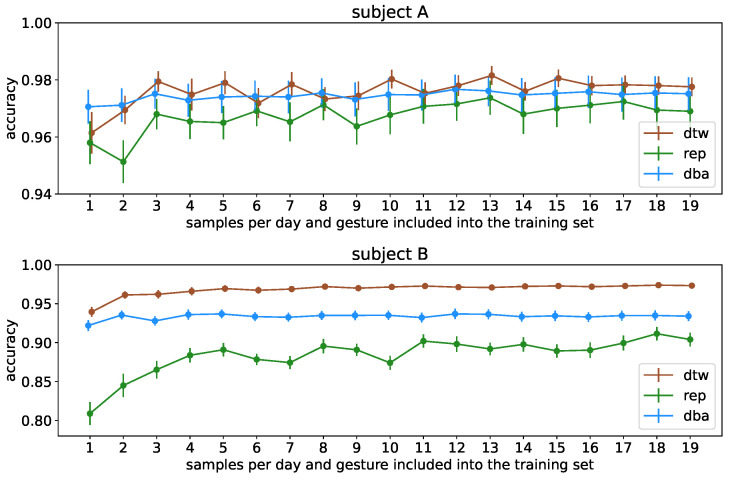
Classification accuracy per subject with different sizes for the training dataset. Based on the five-day-*multiday* dataset, one day was used for the testset, and the remaining four days served for the corresponding training base set. The *x*-axis indicates the number of samples that have been split out into the effective training set per day in the training base set; hence, the total number of samples in the training set is fourfold this value. The data points indicate the average accuracy of the different splits, while the errorbar denotes the range of the standard error on this average.

When investigating constraint effects on the amount of data used for reference as depicted in Figure 9, we find two interesting phenomena: Firstly, we see that the resulting accuracies approach the high level we found initially extremely quickly, converging to final accuracy already after 3 samples per day (i.e., 12 samples total) per gesture for subject A as well as 5 samples per day (i.e., 20 samples total) per gesture for subject B, staying within margins of errors afterwards for the uncondensed as well as the two-step DBA condensed set. We furthermore see more clearly than in Section 3.1 that the one-step condensing method is really insufficient compared to the two-step one, particularly when operating on the multiday dataset, because the representative will ultimately be chosen as a sample on a specific day, i.e., a specific sensor localization, ignoring samples recorded with other seatings of the sensors, whereas the two-step procedure explicitly incorporates them in the final representative sample.

**Figure 10:**
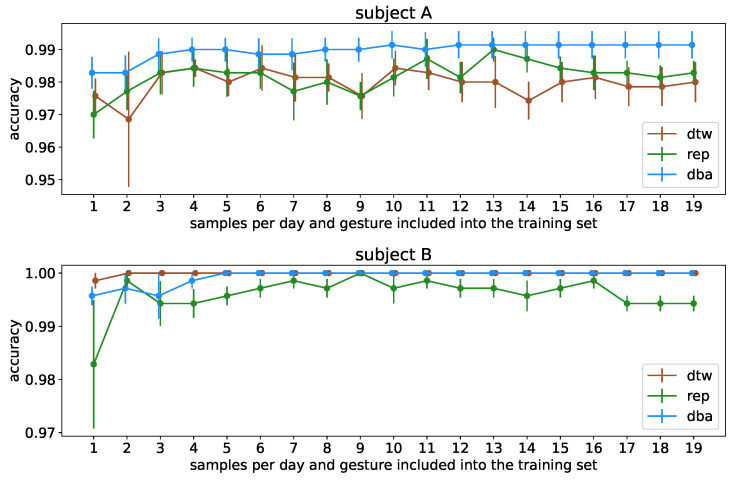
Classification accuracy per subject with different sizes for the training dataset for comparison with Figure 9. Based on the *singleday* dataset, the data were first split randomly into the five-day-*multiday*-like dataset as it is intended as a cross-reference to Figure 9; this artificially constructed 5-day-*multiday* dataset is then processed exactly like the data in Figure 9.

To verify this effect, we contrast this with the same procedure performed on the singleday dataset, artificially split to resemble the multiday dataset but without variability in sensor location and depict Figure 10 as the corresponding equivalent to Figure 9. Here, we clearly see two effects: Firstly, the one-step procedure is especially impacted by the multiday setting due to the effect of not considering sensor location variability which does not occur in the singleday based control setting. At the same time, the two-step barycenter condensing not only is on par with the uncondensed dataset, in contrast to the multiday dataset, it even exceeds the performance of the uncondensed dataset for subject A. This effect can also be observed in the multiday-based investigation for sample sizes of 1 and 2 per day before the uncondensed dataset overall slightly exceeds it in performance. We can conlude that particularly for high-amplitude signals, the barycentering is capable of smoothing out sample-specific artifacts introduced by small sample numbers, whereas this effect is diminished as soon as enough samples are included to guarantee a solid representation of the gesture cluster. We also find that this effect is not reproducible if the signal-to-noise ratio is less advantageous, i.e., for less pronounced muscular physiques.

### 3.4. Acceptance Limitaticons

**Figure 11:**
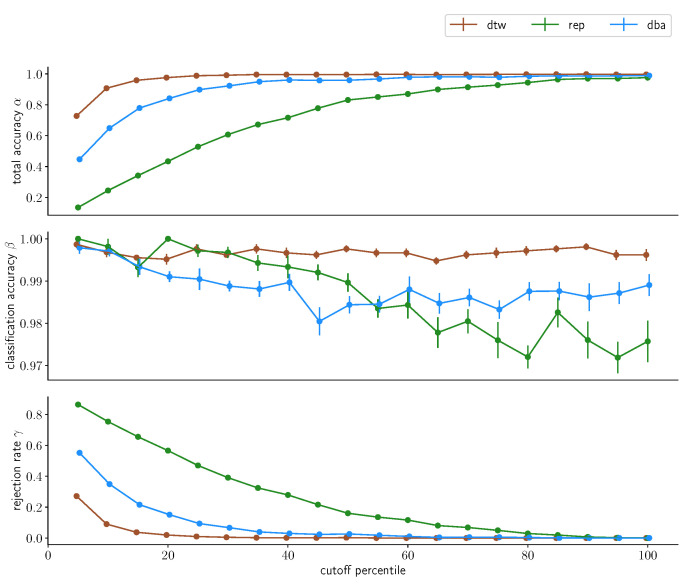
Classification accuracy for different percentiles chosen as cutoff values for the three classification methods for subject A. Classification was performed on the *multiday* dataset, whose individual daily subsets were merged into one large dataset before being randomly split into a reference set containing 70% of the samples and a test set containing the remaining 30% of the samples. This procedure was repeated 10 times. The data points indicate the average accuracy achieved; the corresponding error bars denote the standard error on this average value.

**Figure 12:**
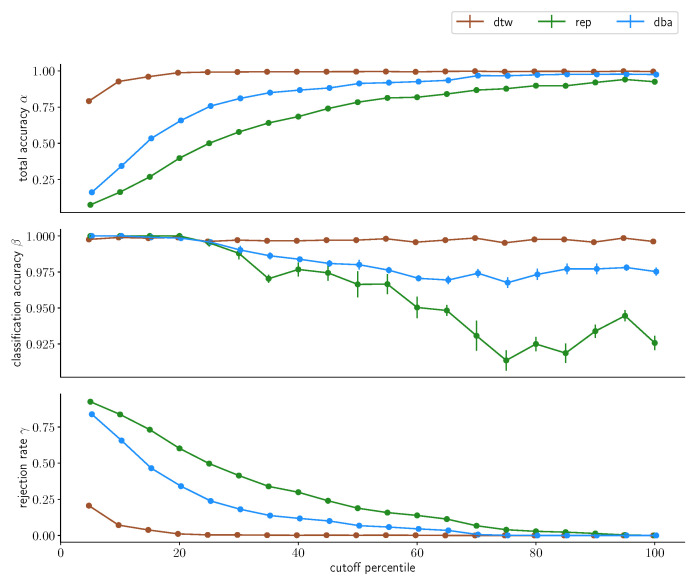
Classification accuracy for different percentiles chosen as cutoff values for the three classification methods for subject B. Classification was performed on the *multiday* dataset, whose individual daily subsets were merged into one large dataset before being randomly split into a reference set containing 70% of the samples and a test set containing the remaining 30% of the samples. This procedure was repeated 10 times. The data points indicate the average accuracy achieved; the corresponding error bars denote the standard error on this average value.

We will now investigate the distance cutoff as percentiles of the intracluster distance distributions we have discussed previously, with the 100th percentile including the full cluster. Firstly, we will take a look at the results derived from merging the multiday dataset into one grand dataset and splitting into reference data and test data, depicted in Figure 11 and Figure 12 for the two subjects. We directly find one observation in the data for subject A that was to be expected: the larger the cutoff distance *d*, the lower the rejection rate γ. This fundamentally holds true for all three approaches, uncondensed, one-step-condensed and two-step-condensed, however with varying quality. While the uncondensed approach barely rejects anything already at *d* being chosen as the 20th percentile, the two-step-condensing method requires about twice the distance, which is, however, unsurprising: While the condensed methods are both one sample that is chosen to resemble the center of a data cluster, the uncondensed approach contains all samples. Hence, *d* gets applied to the outer rim of the cluster in the uncondensed case, whereas it is measured from the center-representing element for condensed methods. Consequentially, the same *d* represents a tighter bound in the condensed cases. This being said, comparing the two approaches we find fundamental differences in performance: while the two-step condensation technique converges rather quickly with respect to the rejection rate, at about the 40th percentile of the cluster distance distribution for the two-step-procedure, the one-step procedure again shows substantially worse performance, barely converging at all, at about choosing the 90th percentile for *d*, again demonstrating that a one-step-condensation is insufficient for strong performance.

For subject B, with the lower signal-to-noise ratio, these results fundamentally hold, the uncondensed approach shows similar convergence results and the two-step-condensation approach takes a little longer for full convergence, still rejecting a little less than a quarter of all samples at *d* being chosen as the 40th percentile but again exhibiting better results than the one-step-condensation approach, again highlighting the preferability.

With regards to classification accuracy β, the uncondensed approach only exhibits minimal changes, if any, and remains at a substantially high accuracy above the 99% mark for both subjects. The two-step-condensation approach degenerates slightly with increasing *d*, but only by about one and two percentage points, respectively, while the one-step-condensation takes a hit of over 8% in accuracy for subject B. The total accuracy overall behaves inversely to the rejection rate, which is expected given the strong performance in classification accuracies $\beta_{s}$, with only minor differences.

**Figure 13:**
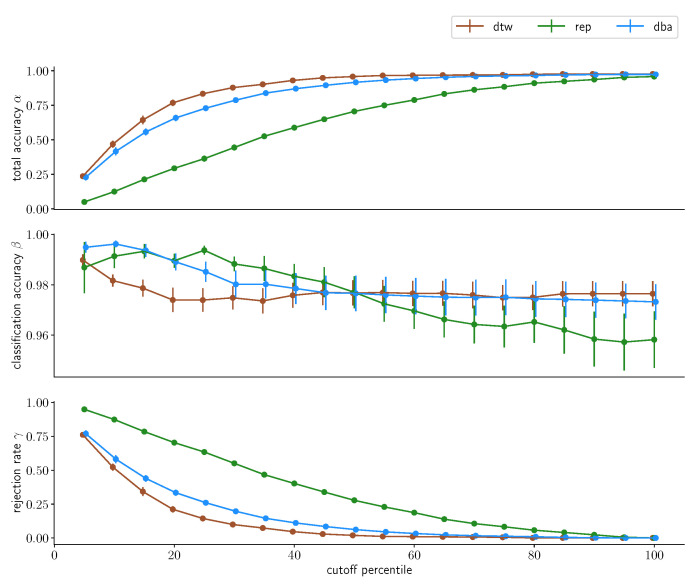
Classification accuracy for different percentiles chosen as cutoff values for the three classification methods for subject A. Classification was performed on the *multiday* dataset with a three/two split for reference and test set, with 10-fold cross-validation. The data points indicate the average accuracy achieved; the corresponding error bars denote the standard error on this average value.

**Figure 14:**
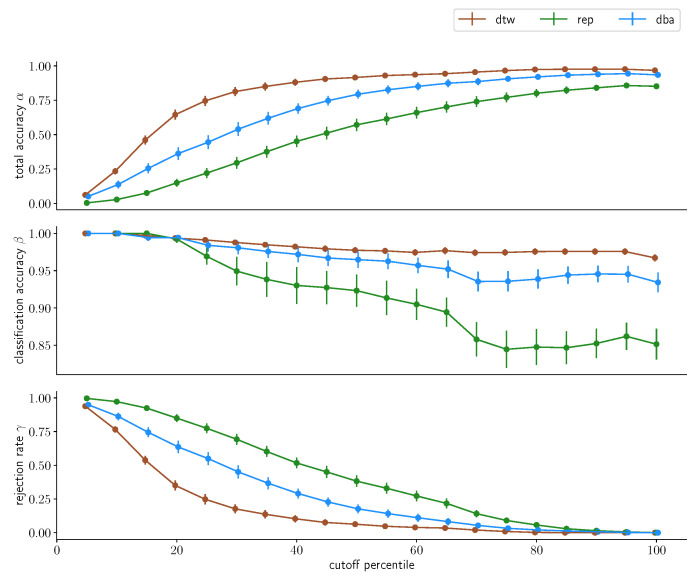
Classification accuracy for different percentiles chosen as cutoff values for the three classification methods for subject B. Classification was performed on the *multiday* dataset with a three/two split for reference and test set, with 10-fold cross-validation. The data points indicate the average accuracy achieved; the corresponding error bars denote the standard error on this average value.

These results do not fundamentally change once we retain the day borders and repeat the process, as depicted in Figure 13 and Figure 14.

When considering a more realistic split by not merging different days, this picture shifts with regard to the uncondensed approach: When day-specific distributions were not retained when constructing the reference data, the uncondensed approach significantly outperformed the other two approaches. Now, when day-local structures are retained, the difference between the uncondensed and the one-step-condensed approach does not vanish but diminishes substantially for very small *d*, whereas the gap starts widening for increasing *d*. On top of that, we find the classification accuracy β of both condensed approaches actually *outperforming* their counterpart $\beta_{uncondensed}$ for subject A up to the 45th percentile for *d* and providing about the same performance for subject B until the 20th percentile before falling off. Still, we find that the two-step-condensation tracks the uncondensed approach in terms of classification accuracy β throughout the spectrum for *d* in this dataset distribution.

This indicates that while the condensed approaches exhibit a stronger performance degradation on an ideal dataset, the two-step-condensation technique exhibits comparable performance to the uncondensed approach for a more realistic dataset configuration that respects the localization noise inherent in an everyday use scenario. Especially the consistency with regards to the classification accuracy indicates that while the distinction might be drawn between “high accuracy, high rejection” and “low accuracy, low rejection”, this is not actually as substantial as one might have expected in the beginning. Instead, we find a rather consistent classification accuracy over most of the spectrum, with slightly higher resulting β for very small *d*, but we find a strongly increasing total accuracy and fast-dropping rejection rate with increased *d*. So, if the application is not critical and misclassifications do not have to absolutely be avoided (if that is the case, one should err on the side of very small *d*, increasing the rejection rate but ensuring highly accurate assessments of input gestures), it seems that some leniency on *d* will barely compromise on resulting classification performance β while reducing the rejection rate γ substantially, which would be particularly suited for non-critical applications.

### 3.5. Inter-Participant Assessment

**Figure 15:**
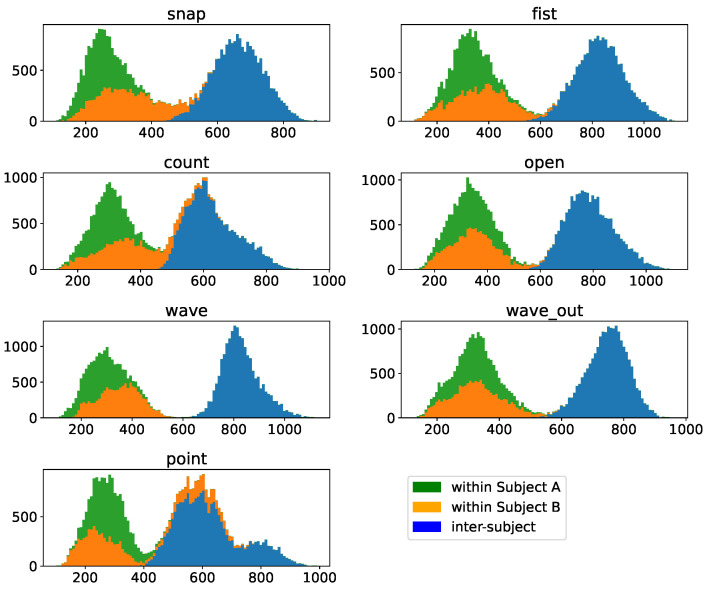
Pairwise DTW-distances of clusters per gesture within- as well as inter-subject based on unnormalized samples. The within-subject distances are pairwise distances of samples of the denoted gesture with all other samples of this sample as well as subject. The inter-subject data indicate pairwise distances between samples of the same gesture but different subjects. The total histogram consequentially indicates the pairwise distances per gesture after merging the sample sets of both subjects.

Finally, we will assess how well we can transfer data between participants, which is, should it work, extremely useful in kickstarting the usability of such a system. To do so, we will compare the intracluster distances $\delta_{s}$ for the same gesture for each participant with the intercluster distribution Δ between the same gesture of two participants, the results of which are depicted in Figure 15. The resulting distribution is clearly bimodal, and the bimodality is, with a slight exception for the “point” gesture of subject B, completely dominated by separating the intercluster distance distribution Δ from the intracluster distance distributions $\delta_{s}$. While this fact alone does not yet make the approach unusable, we can take a look at the numbers of the Dynamic Time Warping distance and compare them to the gesture-comparison distributions in Figure 5 and Figure 6. We find that the intercluster distribution Δ between the same gesture of subjects is centered at about or only slightly lower DTW distances compared to the intercluster distance distributions between gestures of the same subject. This implies that clusters of the same gesture of our two participants are about as far apart as clusters of different gestures within one subjects is, posing a substantial challenge to the direct transfer between subjects.

To address this effect, we will now investigate whether sensor-specific or batch normalization to muscular strength as described in Section 2.2.6 will remedy this issue by addressing the different physiologies of the participants. To generate the normalization factor, subjects were asked to record an additional gesture type, albeit in lower freqeuncy, which we refer to as “powergrip”. They were instructed to grasp an object within their palm and to exhibit maximum voluntary contraction of all muscles by gripping as tightly as possible over a timeframe that they can hold this maximum voluntary contraction. We subdivide these recordings into three equally large sub-intervals, take the center third of each recording and average all data points over time to obtain an average normalization factor for both sensor-specific as well as batch normalization. We exclude the first third to conservatively ensure not to include any ramp-up to maximum voluntary contraction and we exclude the last third to avoid including data where the subject might be inadvertently tiring out their muscles for the current powergrip recording before they stop.

**Figure 16:**
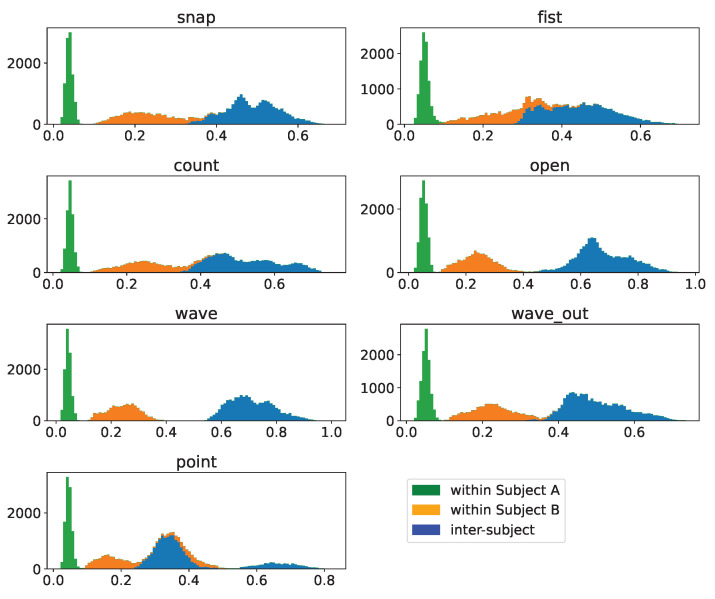
Pairwise DTW distances of clusters per gesture within- as well as inter-subject based on batch-normalized samples. The within-subject distances are pairwise distances of samples of the denoted gesture with all other samples of this sample as well as subject. The inter-subject data indicate pairwise distances between samples of the same gesture but different subjects. The total histogram consequentially indicates the pairwise distances per gesture after merging the sample sets of both subjects.

**Figure 17:**
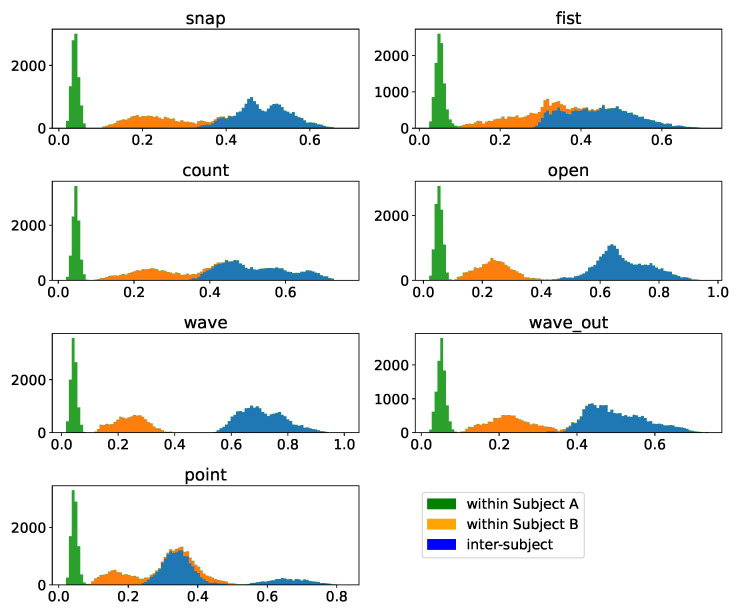
Pairwise DTW distances of clusters per gesture within- as well as inter-subject based on sensor-specifically normalized samples. The within-subject distances are pairwise distances of samples of the denoted gesture with all other samples of this sample as well as subject. The inter-subject data indicate pairwise distances between samples of the same gesture but different subjects. The total histogram consequentially indicates the pairwise distances per gesture after merging the sample sets of both subjects.

We then re-perform the aforementioned comparison, the results of which can be seen in Figure 16 and Figure 17. Three things can be observed by looking at these results: Firstly, we find no substantial difference between batch and sensor-specific normalization to the point that they are almost indistinguishible, with existing, but in absolute terms miniscule differences. Secondly, while the distribution in the unnormalized case was bimodal, with the two within-subject intracluster distributions δ stacking, we now see a separation between these two. The reason for that is the different signal-to-noise ratios of the subjects: While subject A exhibits a higher signal amplitude at the same level of noise and subject B’s amplitude at the noiselevel of the measurement hardware is lower, the normalization factor differently affects the noise level, as the normalization factor is dominated by the signal amplitude. Hence, the larger normalization factor for subject A will reduce the noise level more strongly, leading to a substantially more narrow distribution, whereas the distribution for subject B, which exhibits a lower signal amplitude, is not driven down as much as a consequence, resulting in a less narrow intracluster distribution at higher distance levels. Finally, and most importantly, this also does not lead to a reduction in separation between the intra- and intercluster distances, hence leaving the question of transferability between subjects still open. While we cannot state that there is no way of achieving this, this simple approach certainly seems to be insufficient for the task. This is supported by [22], who reported a degeneration of accuracy from 89.5% down to 53.7% when including inter-participant generalization in their model by merging the reference data of multiple subjects into one reference set and testing with a subject that had not been included. This degeneration is consistent with our finding that the inter-participant DTW distances of the same gesture are in the same magnitude as the inter-gesture distances within one participant, explaining this steep plunge in accuracy.

## 4. Discussion

In the introduction, we asked three key questions that we can now confidently answer:

*Is the classification accuracy sufficient for use of such a system in practice?*

With classification accuracies solidly above 97%, this can be confirmed clearly and straightforwardly, both for the uncondensed approach as well as for the more computationally efficient, condensed approaches, especially the DBA condensation.

*What is the underlying reason this classification exhibits this classification performance?*

The relative pointwise distance distributions between and within the gesture clusters, depicted in Figure 5 and Figure 6, show a clear distinction between the 90th percentile of the intracluster distance distribution and the next-closest 10th percentile of the intercluster distributions for most combinations, with the upper end of the intracluster distance distribution, which could be perceived as an approximation of cluster diameter, being smaller than the lower end of the intercluster distance distribution, which can be perceived as approximating the border-to-border distance between the clusters. Due to the cluster diameters being solidly smaller than the distances to the next cluster, samples of different clusters typically separate nicely in the distance space we are looking at in this work.

The only exception are the *wave out* and *point* gestures, but while we see a case of weaker separation, the separation is still sufficient to not impede the classification. It should be remembered that the intracluster distribution characterizes the diameter of the cluster and not its radius and hence the comparison between the upper limit of the intracluster distance distribution with the lower limit of the intercluster distance distribution is still a rather conservative measure, hence the negligible impact on classification performance.

*How can this classification performance be retained reducing both computational and user effort?*

We have shown two approaches for reducing the computational cost by condensing gesture clusters onto fewer samples, in our specific case one single sample per cluster. While the reduction in computational cost through this approach is easily evident, we could also show that especially the condensation approach computing a single barycenter sample as the cluster representation does exhibit an accuracy impact in most cases, but it typically ranged about 1%, depending on the exact investigation at hand. Especially in comparison with the accuracy readings we find both in our method and the current state of the art, this seems to be quite an acceptable tradeoff.

More generally, the results we have shown outline the usability of the approach we are proposing, particularly with an emphasis on structural understanding of how the recorded samples are distributed. This allowed our approach not only to exceed the classification accuracies that are available in comparable work but also allowed the derivation of follow-up methods that naturally emerge from the structural findings. Of particular relevance are structure-respecting condensation methods that allow a substantial reduction in overall computational cost while retaining high performance or in some situations even exceeding the performance of the uncondensed dataset. We have, however, seen that not all condensation methods work equally well, so although structure can inform an understanding, we still need to make use of this understanding to propose methods that yield beneficial performance. In this context, the two-step barycenter averaging procedure was one of those. The investigation and consequential exploitation of underlying structures also made this approach extremely robust with regards to the number of samples required, yielding very good performance even with as low as single-digit reference samples for everything that shall be detected. This strong accuracy can possibly even be improved further as we have seen that some of the gestures in the set we picked are easier to distinguish from one another than others, and while we have not reduced the dataset as a consequence of this for this particular investigation, it shows a clear and intuitive approach of how to assess which gestures are well separable and which might be not as separable and hence not ideal to be combined in a gesture control system. We can, in fact, use that to derive an approach of picking a set of a specific number of gestures from a larger gesture set that is specifically tailored to good separability and consequentially little misclassification in a gesture control system. The observations in Section 3.2 intuitively yield a systematic quantification of the quality of a gesture set with regards to its differentiability: When deciding on a set $\{g_{i}\}\in G$ of *n* gestures from a pool of *m* gestures *G*, one wants to prefer those gestures that maximize the distance to all other gestures in the chosen set, such as

$$\begin{array}{cccc} \; & \underset{\left\{g_{i}\right\}}{\mathrm{minimize}}\; & \; & ||10th\left(\Delta_{g_{i},g_{j}}\right)-90th\left(\delta_{g_{i}}\right){\left|\right|}_{\infty }\\ \; & \mathrm{subject}\;\mathrm{to}\; & \; & \{g_{i}\}\subset G\\ \; & \; & & \#\{g_{i}\}=n\;. \end{array}$$

This way, a user can even simply pick a gesture set themselves and individualize the system that way. By providing more gestures than necessary to cover all actions one wants to trigger, such an approach could automatically propose the best combination of gestures the user individually provided, making the system very usable in personal praxis.

Such an approach also assists as our current investigations have not yet uncovered an approach that reliably allows transfer of recordings between participants. This problem has two fundamental contributions: Firstly, the muscular physique between participants differs, and hence the muscle signature of the individual gestures might not be identical. This, however, seems to be more of an overall physique problem than a different strength of individual muscle groups, as batch and sensor-specific normalizations would have exhibited a larger difference in resulting distance distributions. This is, however, also challenged in addition by the fact that the electrodes applied by the Myo hardware are not targeting specific muscle groups but instead equidistantly spread the electrodes around the forearm at the same height, capturing a good overall picture of muscle activity at that position, but this positioning is likely limited in differentiating between muscle groups due to a lack of muscle-specific targeting. While it seems unlikely that this can be resolved as long as such a system is supposed to be operated by a user on their own, a comparative dataset that targets specific muscle groups would establish an interesting baseline to quantify how large the impact of this equidistant ring-placement of sensors actually is. This would also be of interest with regards of whether participant transfer is fundamentally limited, limited for the specific sensor arrangement at hand or especially limited for the two participants specifically currently included at this point. Furthermore, for additional studies, a quantification of the level of training, either by measuring circumference of the forearm at placement position or other methods of establishing a quantification of muscle mass, should be obtained. While the insight of this might be limited in a two-participant study, it will certainly be of interest once the number of participants is increased.

Secondly, for this particular investigation, users were instructed how to perform the gesture set and it was ensured that the instructions were understood, but it was not controlled how they were executed exactly to reproduce the situation as it would emerge if someone privately used such a gesture recognition system. Hence, variations in exact execution could also add to the individuality of muscle signatures. A comparative study with supervised recordings in addition to a purely unsupervised setting might be able to shed light on this specific question.

## 5. Conclusions

We have shown that, leveraging Dynamic Time Warping and a k-Nearest-Neighbors classifier, we can classify gestures purely on their muscle signature with extremely high accuracy. We were able to retain this accuracy while also reducing computational cost by selecting or generating representatives for target gestures. Furthermore, it could be shown that even though geometric arguments can no longer be made, a geometric analogy can be established, allowing for an intuitive understanding of how these high accuracies are emerging, which sets this approach apart from commonly used black-box approaches that do not facilitate an inherent understanding of the underlying mechanisms of the method.

This directly translates into an intuitive ability for establishing different properties in this method: Both the constrainment on the amount of reference data necessary and even more so the introduction of an intuitive approach to differentiate the measured data from any form of continuous background stream or unrelated data classes could intuitively be constructed and their effects intuitively understood, which helps in the directed development of such a method while also gaining further insight into the systematic structure of the underlying data.

This also allowed for an intuitive understanding of how to employ inter-subject analysis, although the data so far has proven to be insufficient for this to be a viable use case in practice. There are three potential reasons for this that come to mind, that probably all play into it: Firstly, the recording of maximum voluntary contraction was not appropriately representing the necessary normalization for this transfer approach to work. Secondly, even with correct normalization in place, the different muscle structures of the participants might contribute to different signal reads, particularly considering differing signal-to-noise ratios. Finally, and potentially the most significant: The fundamental goal of this study was to obtain data in the context of a realistic application. Hence, participants were instructed but not supervised when recording the different gestures. Consequentially, individual variations might change the gesture muscle signatures enough between participants to make a transfer practically unviable.

To investigate this further, it would be interesting to obtain a more supervised collection of data to minimize the impact of individual variations in performing the gestures. This might shed light on the contribution of these three options to the non-viability of the transfer. An effective transfer from one user to another would further alleviate the need to record a reference dataset, making the time from introduction to operation even smoother.

Nevertheless, as it has been shown that the number of reference samples can be significantly smaller than the reference datasets we acquired while still preserving the presented high accuracies, this approach is still practical as long as the system is set up with such subject-specific data before usage. An analysis similar to [20] could also be additionally helpful to identify gestures that allow for multisubject use of reference recordings.

This is also an excellent result with regards to further data acquisition. While two participants have been sufficient to demonstrate the fundamental viability of the proposed method, it will be interesting to see how it fares with more participants, especially when we investigate participant transfer of data. What we have seen so far is that, specifically when targeting the question of participant transfer, precise thought needs to be put into what physiological information can be obtained to correct EMG signals for successful participant transfer and to find out what is necessary to make such a transfer work and if that effort is ultimately viable for an application system.

## Figures and Tables

**Table 1:** Number of seatings and samples acquired for this investigation.

Dataset	Singleday	Multiday
number of seatings	1	5
samples per seating per gesture	100	20
samples per seating total	700	140

**Table 2:** Dataset splits for different analyses.

Analysis Type	Dataset Used	Dataset Split (Reference/Test)	Cross-Validation Folds	Results
Fundamental accuracy analysis	singleday	70%/30%	400 per graph row	Section 3.1
Relative cluster distributions	multiday	-	-	Section 3.2
Constraining reference requirements	both	4 days/1 day	10 per point	Section 3.3
Acceptance distance limitations	both	70%/30% & 3 days/2 days	10 per point	Section 3.4
Inter-participant distance distributions	singleday	-	-	Section 3.5

## Data Availability

For reasons of data privacy, the data recordings cannot be published alongside this manuscript. Please contact the authors for questions of availability.

## Abbreviations

The following abbreviations are used in this manuscript:

DTW	Dynamic Time Warping
kNN	k-Nearest-Neighbors
DBA	DTW Barycenter Averaging
